# Micro-competition system for Raman quantification of multiple glycans on intact cell surface†
†Electronic supplementary information (ESI) available: Experimental details and supplementary figures. See DOI: 10.1039/c5sc01031d



**DOI:** 10.1039/c5sc01031d

**Published:** 2015-04-30

**Authors:** Yunlong Chen, Lin Ding, Junqiang Xu, Wanyao Song, Min Yang, Junjie Hu, Huangxian Ju

**Affiliations:** a State Key Laboratory of Analytical Chemistry for Life Science , School of Chemistry and Chemical Engineering , Nanjing University , Nanjing 210093 , P.R. China . Email: hxju@nju.edu.cn ; Fax: +86 25 83593593 ; Tel: +86 25 83593593; b Department of Pharmaceutical & Biological Chemistry , UCL School of Pharmacy , University College London , London WC1N 1AX , UK

## Abstract

A micro-competition system is designed for simultaneous quantification of multiple glycans on intact cell surfaces, by integrating two-surface–one-molecule competition with surface enhanced Raman scattering (SERS). The micro-competition is achieved among multiple-polysaccharide-coated gold nanostars functionalized silica bubbles, target cells and gold nanoprobes at a micron scale. The gold nanoprobes are prepared by coating distinct Raman molecules and lectins on gold nanoparticles for signal resolution and glycan recognition, respectively. The silica bubble surface serves as an artificial glycan surface and a SERS substrate. Upon the competitive recognition of lectin to the corresponding glycan, the gold nanoprobes can be specifically captured by the bubbles and cells in a homogeneous system, and the amounts of different gold nanoprobes on bubbles are simultaneously detected by SERS to reflect the corresponding glycan amounts on the cell surface. This micro-competition system with multiple quantification capability provides a powerful tool for investigation of the complex glycan-related biological processes.

## Abstract 2

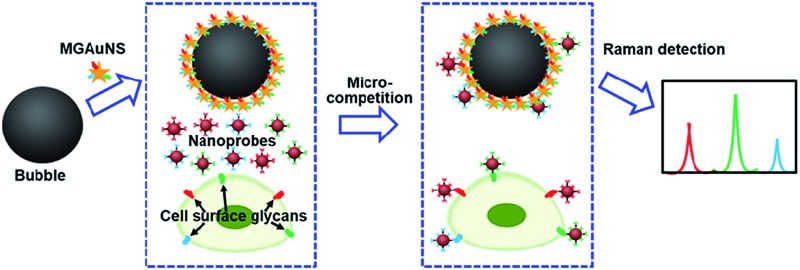
A micro-competition system integrated functionalized silica bubbles and Raman encoded nanoprobes to simultaneously assay multiple glycans on intact cell surfaces.

## Introduction

Glycans, which decorate all mammalian cell surface, mediate a wide variety of biological processes, such as cell communication, immune response, pathogen interaction and intercellular signaling events.^1,2^ Their structure complexity and microheterogeneity can dynamically reflect the physiological and pathological states of cells.^3–7^ Aberrant glycosylation of proteins and aberrant glycan expression on cells have been associated with many diseases such as cancer. The terminal glycan motifs can promote invasive behaviour of tumour cells that ultimately leads to the progression of cancer.^8^ Therefore, simultaneous analysis of multiple glycans on living cell surfaces shows great importance in research into the correlation between the specific glycan patterns and their roles in disease states and developments.

Regarding multiplexed glycan detection, mass spectrometric analysis is a powerful tool for providing molecule-level information,^9^ but it is unsuitable for *in situ* detection, and suffers from the undervaluation of certain glycans during the tedious cell lysis, enzymatic cleavage and derivatization processes.^10^ Alternatively, encoding-based lectin array techniques have been developed for multiplexed glycan profiling.^11–13^ However, those protein immobilization based methods suffer from the denaturation or inactivation of proteins, due to chemical modification and spatial inaccessibility on arrays.^14,15^ To overcome these disadvantages, our previous work designed barcode-lectin probes for *in situ* fluorescence analysis of multiple cell surface glycans through a DNA microarray for decoding.^16^ In fact the preparation of barcode-lectin probes is relatively expensive and time consuming. Thus it is necessary to develop novel economic and practical coded probes for quantification of multiple glycans on intact cell surfaces.

Besides the fluorescence based technique,^17^ surface-enhanced Raman scattering (SERS) has also been used for evaluation of single cell surface glycans.^18^ This method cannot give quantitative results. In view of the advantages of SERS that can provide the complete vibrational information of molecules,^19,20^ and Raman signal molecules (RSMs) which can be used to code for multiplexed detection,^21–23^ we prepared a set of Raman barcoding probes to couple with a newly designed micro-competition system for fast, quantitative detection of multiple glycans on cell surfaces.

**Fig. 1:**
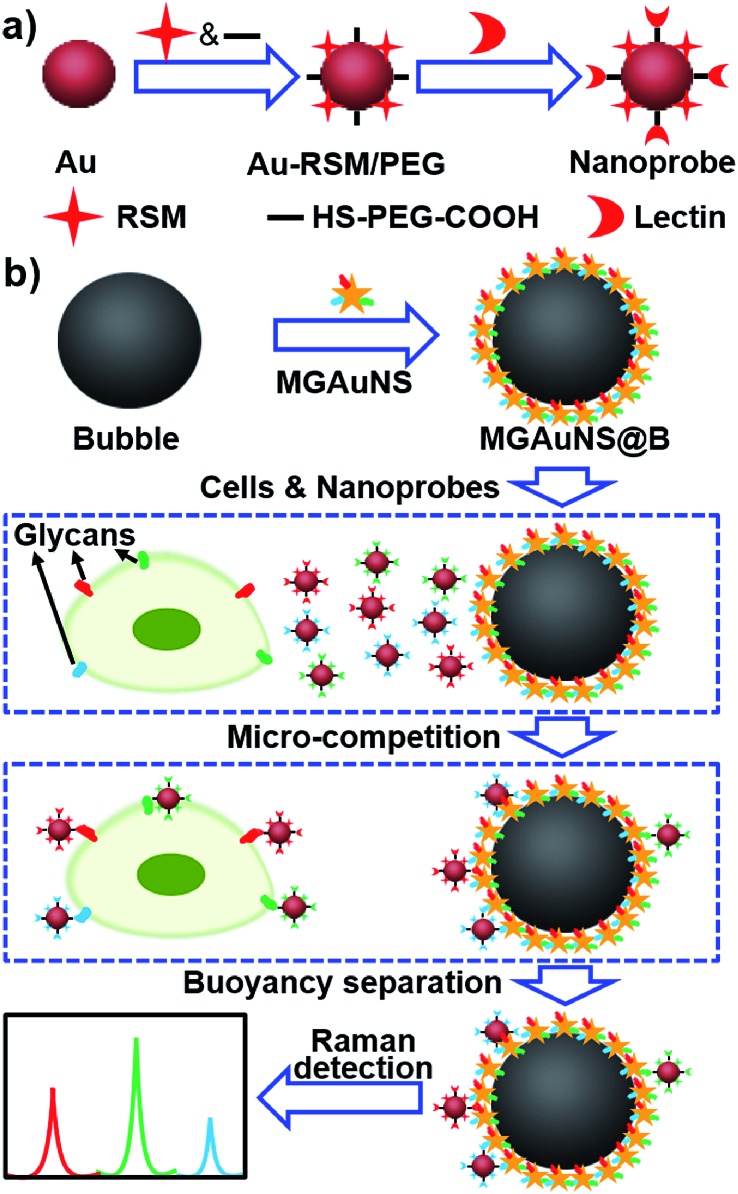
Schematic illustration of (a) synthesis of nanoprobes and (b) micro-competition system for multiple Raman detection of cell surface glycans.

Competition is an ingenious quantification method, which can eliminate virtually any chance of false positive background signals. The micro-competition system consisted of three components: Raman signal molecule and lectin dual-coded gold nanoparticles (AuNPs), represented by nanoprobes, silica bubbles functionalized by multiple-polysaccharide-coated gold nanostars (MGAuNS@B), and cells. Nanoprobes were respectively coded with three kinds of RSMs with distinguishable characteristic peaks, and subsequently functionalized with different lectins to specifically recognize target glycans (Fig. 1a). The AuNSs are powerful SERS substrates owing to the strong plasmonic electromagnetic field due to their anisotropic nanostructure.^24–27^ The MGAuNS@B were prepared by *in situ* coating multiple polysaccharides on AuNS surfaces, and stepwise assembling the coated AuNSs onto silica bubbles by electrostatic adsorption. After mixing MGAuNS@B, nanoprobes and cells in PBS, the designed anisotropic multiple glycan surface could compete with cell surface glycans for binding different types of nanoprobes, *via* lectin–glycan recognition in a one-molecule–two-surface format at a micron scale, thus called a micro-competition system. Following completion of binding, MGAuNS@B could be quickly separated by buoyancy^28–30^ and the amount of bound nanoprobes could be detected by Raman spectrometry (Fig. 1b).

Compared with the complanate glycan surface previously designed for competitive recognition,^31–34^ the anisotropic surface of MGAuNS@B with dimensions close to the cells provided a better simulation of cell surface glycans for more efficient competitive recognition.^35,36^ Moreover, the MGAuNS@B could act as SERS substrates for nanoprobes that could not generate Raman signals individually due to the small size. The amounts of glycans on cell surfaces could be accurately reflected by the amounts of nanoprobes selectively bound on the artificial glycan surfaces for sensitive Raman detection. The proposed glycan profiling method possesses whole-surface accessibility, rapid detection, enhanced sensitivity, high throughput and the advantage of cost effectiveness.

## Results and discussion

### Characterization of nanoprobes and MGAuNS@B

**Fig. 2:**
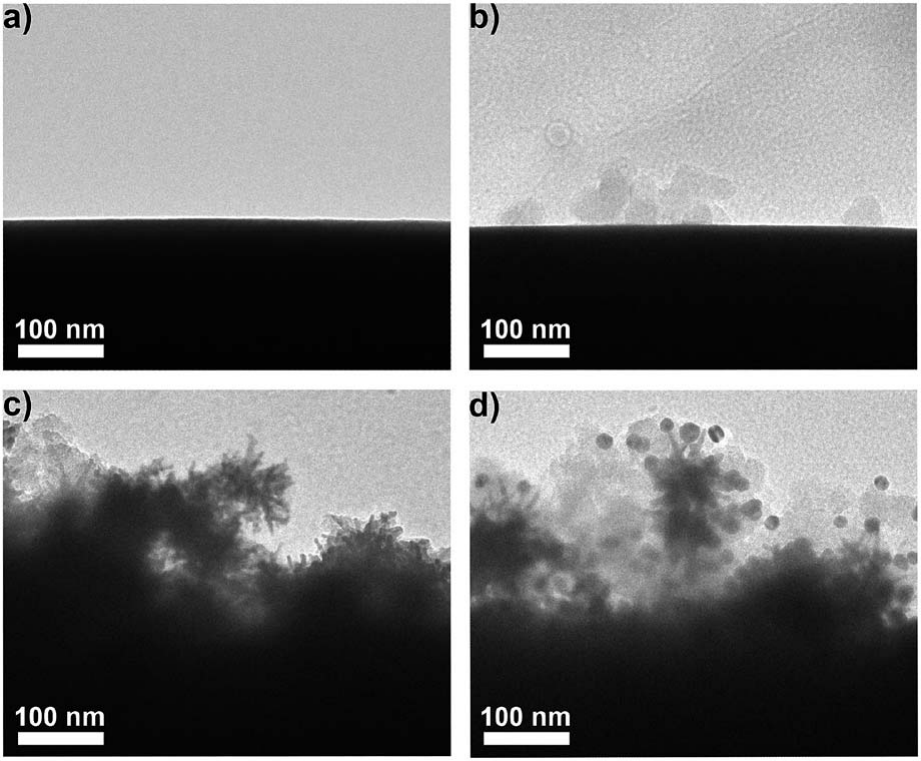
TEM images of (a) bubble, (b) PDDA-bubble, (c) MGAuNS@B, and (d) nanoprobes-bound MGAuNS@B.

AuNPs were firstly modified with sulfhydryl contained polyethylene glycol (PEG) and subsequently coded with RSMs through Au–S binding and lectins *via* EDC-mediated carbodiimide chemistry. Three pairs of RSMs and lectins were involved: 2-naphthol (NT) and *Lens culinaris* agglutinin (LCA), 4-aminothiophenol (ATP) and *Sambucus nigra* agglutinin (SNA), and 5,5′-dithiobis(2-nitrobenzoic acid) (DTNB) and succinylated wheat germ agglutinin (SWGA). LCA and SNA can specifically recognize mannose (Man) and *N*-acetylneuraminic acid (Sia) respectively (Table S1†), while SWGA cannot bind to sialic acid residues but retains its specificity toward *N*-acetylglucosamine (GlcNAc) (; http://www.vectorlabs.com/catalog.aspx?catID=253). The UV spectra showed the characteristic peak of proteins at 280 nm, indicating the efficient binding of lectins (Fig. S1†). Polysaccharide-coated AuNSs were synthesized by directly adding three kinds of polysaccharides to the growing solution of AuNSs. TEM images illustrate an obvious sugar layer on the AuNSs compared with AuNSs without polysaccharide coating (Fig. S2a and S2b†). Zeta potential measurements showed a more negative charge and more narrow size distribution of AuNSs after polysaccharide coating (Fig. S2c†), which was possibly owing to the stability and protection of polysaccharides in the one-pot synthesis. The polysaccharide coating did not affect the SERS ability of AuNSs, as verified by the almost unchanged Raman signal intensity after sugar coating (Fig. S2d†). Since the polysaccharide-coated AuNSs were negatively charged, they could adsorb to the positively charged poly(diallyldimethylammonium chloride) (PDDA)-coated silica bubbles. TEM images illustrate an obvious morphology change with the step-by-step assembly of MGAuNS@B (Fig. 2a–c). After incubation with the mixture of three kinds of nanoprobes, the MGAuNS@B surface displayed the bound nanoprobes (Fig. 2d).

**Fig. 3:**
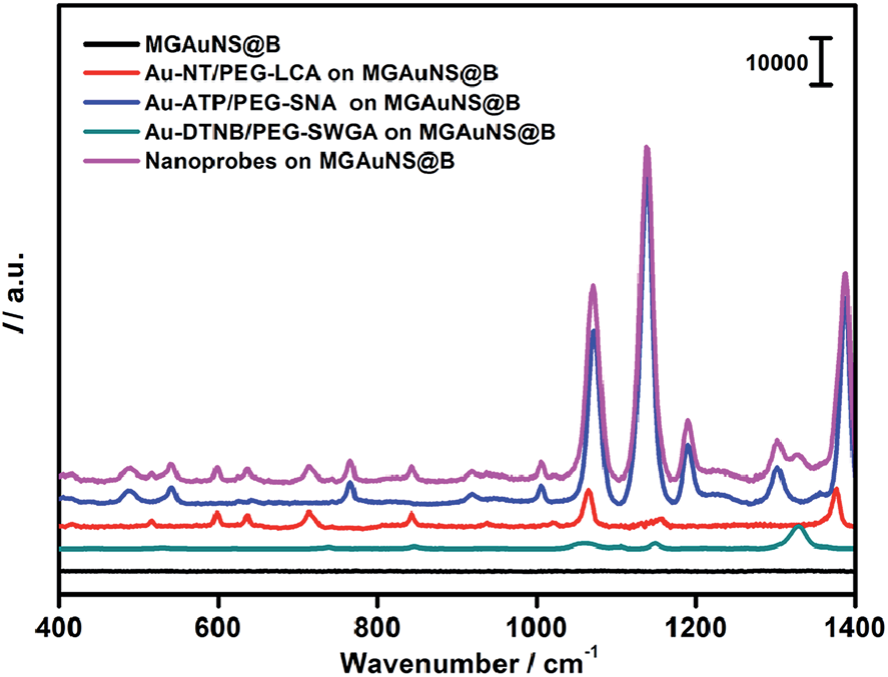
Raman spectra of MGAuNS@B before and after incubation with three nanoprobes and their mixture.

To verify the feasibility of Raman coding, MGAuNS@B was incubated with either a single type of nanoprobe or a mixture of three types of nanoprobes. MGAuNS@B did not show any Raman response, while the nanoprobes showed the characteristic Raman peaks of the corresponding RSM (Fig. 3). The nanoprobe mixture also showed the characteristic peaks at 715.5 cm^–1^, 1189.7 cm^–1^ and 1328.6 cm^–1^, indicating an easy-to-observe distinction on the overlay spectrum. These peaks were chosen as the Raman barcode peaks for detection of three glycans on intact cell surfaces.

### Recognition specificity

The binding specificity of the nanoprobes to MGAuNS@B was verified with three types of silica bubbles, which were functionalized with different single polysaccharide-coated AuNSs. After incubation with the mixture of three nanoprobes, these bubbles could only bind the corresponding nanoprobe, while the bubbles modified with bare AuNSs did not showed any binding to the nanoprobe (Fig. S3†).

The recognition specificity was further validated by monosaccharide inhibition testing (Fig. S4†). After the mixture of nanoprobes was pre-inhibited with Man, Sia, or GlcNAc for 2 hours, it was incubated with MGAuNS@B. The Raman spectra of the resulting bubbles did not show the Raman barcode peak of the nanoprobe corresponding to the monosaccharide, while the Raman peaks coded with other lectins increased with the increasing concentration of the nanoprobes, indicating the specific inhibition of the nanoprobes by the corresponding monosaccharides with negligible recognition from the three recognition pairs.

### Quantitative detection of multiple cell surface glycans

To achieve highly sensitive detection, the optimal time of binding between nanoprobes and MGAuNS@B was determined to be 60 minutes, at which the competitive binding of nanoprobes to cells and MGAuNS@B could be completed (Fig. S5†).

**Fig. 4:**
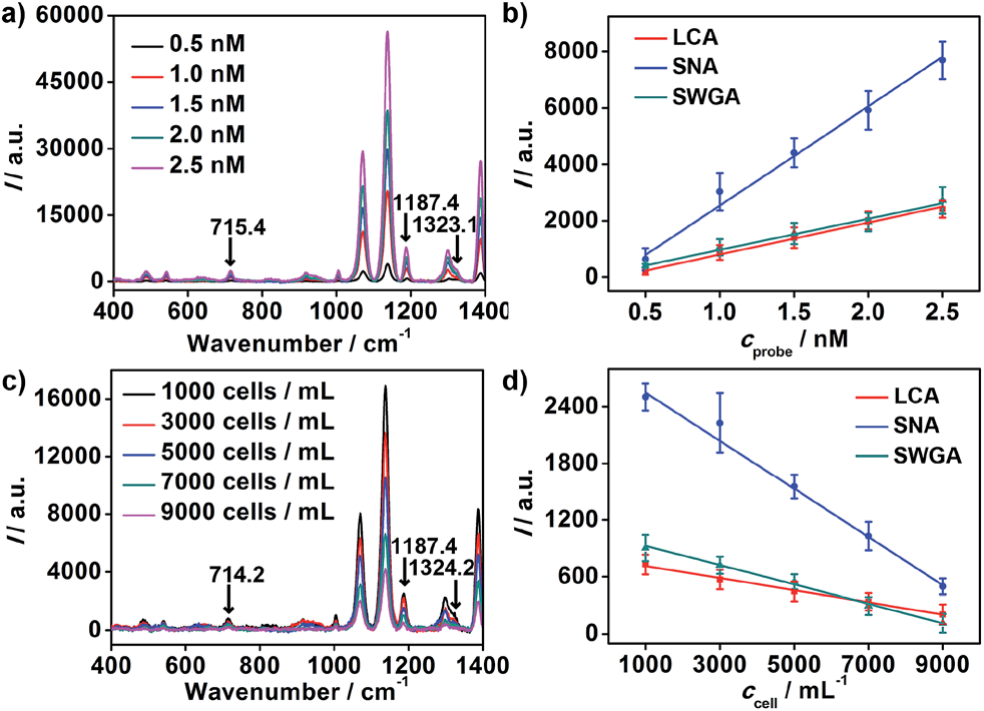
(a) Raman spectra of MGAuNS@B after incubation with the nanoprobe mixture at different concentrations. (b) Plots of Raman intensity at 715.4 cm^–1^, 1187.4 cm^–1^ and 1323.1 cm^–1^ *vs.* nanoprobe concentration. (c) Raman spectra of MGAuNS@B after competition with different MCF-7 concentrations. (d) Plots of Raman intensity *vs.* cell concentration.

With the incubation time of 60 minutes, the standard binding curves of nanoprobes with MGAuNS@B are shown in Fig. 4a and b. For each kind of nanoprobe, the Raman intensity (*I*) of MGAuNS@B at the corresponding barcode peak was proportional to the nanoprobe concentration (*c*_probe_) in the range from 0.5 to 2.5 nM (Fig. 4b):

1 *I* = *k*_1_*c*_probe_ + *b*_1_

In the presence of MCF-7 cells, the nanoprobes were competitively captured by the cells. The concentrations in the incubation mixture and MGAuNS@B were the total concentration (*c*_probe0_) –*a*_probe on each cell_ *c*_cell_, where *a*_probe on each cell_ is the amount of nanoprobe bound on each cell. Thus

2 *I* = *k*_1_(*c*_probe0_ – *a*_probe on each cell_*c*_cell_) + *b*_1_

*i.e.*

3 *I* = –*k*_1_*a*_probe on each cell_*c*_cell_ + (*b*_1_ + *k*_1_*c*_probe0_)

At a constant *c*_probe0_ of 1 nM, *I* was inversely proportional to MCF-7 cell concentration (*c*_cell_) in the range from 1.0 × 10^3^ to 9.0 × 10^3^ cells per mL (Fig. 4c and d):

4 *I* = *k*_2_*c*_cell_ + *b*_2_

From eqn (3) and (4):

5 *a*_probe on each cell_ = –*k*_2_/*k*_1_

Hence, the amount of different nanoprobes bound on each cell could be calculated, which could be regarded as the amounts of the corresponding glycan on each cell.

The slopes of the standard binding curves were determined as 1.1 × 10^3^, 3.5 × 10^3^ and 1.1 × 10^3^ for LCA, SNA and SWGA coded nanoprobes, and the slopes of MCF-7 cell competition curves were –0.064, –0.26 and –0.10, respectively, for the corresponding nanoprobes. According to eqn (5), the average numbers of MCF-7 cell surface Man, Sia and GlcNAc could be calculated as 3.4 × 10^7^, 4.4 × 10^7^ and 5.5 × 10^7^ per cell. The relative expression extent was in good agreement with the results from flow cytometric analysis using fluorescein-labeled lectins (Fig. S6†), demonstrating the feasibility of the proposed method for simultaneous detection of multiple glycans on living cells.

**Fig. 5:**
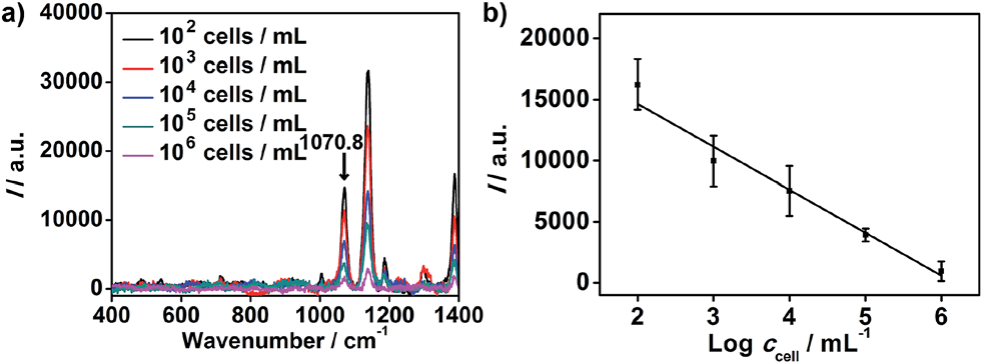
(a) Raman spectra of MGAuNS@B after competition with MCF-7 cells of different concentrations. (b) Plot of Raman intensity at 1070.8 cm^–1^ shown in Fig. 3 *vs.* cell concentration.

The proposed strategy could also be used for cell quantification. The Raman characteristic peaks of three RSMs overlaid at 1070.8 cm^–1^ (Fig. 3), so the peak intensity at 1070.8 cm^–1^ could be used for quantification of cells. *I* at 1070.8 cm^–1^ was found to be inversely proportional to logarithmic cell concentration in the range from 10^2^ to 10^6^ cells per mL (Fig. 5). Considering that the volume of cell suspension in the micro-competition system was 100 μL, the proposed method could realize a detection limit down to 10 cells.

### Monitoring of multiple cell surface glycans

**Fig. 6:**
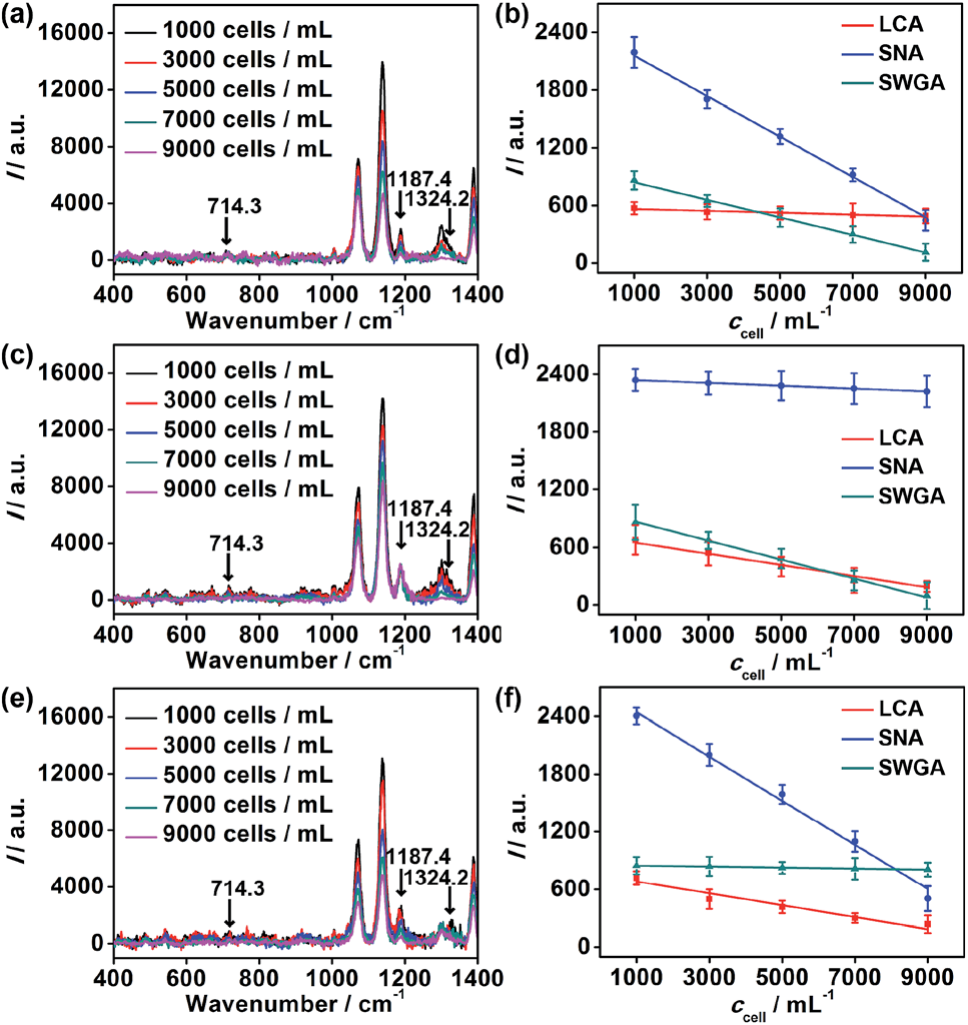
Raman spectra of MGAuNS@B after competition with (a) mannosidase, (c) neuraminidase or (e) *N*-acetyl-glucosaminidase-treated cells of different concentrations. Plots of Raman peak intensity at 714.3 cm^–1^, 1187.4 cm^–1^ and 1324.2 cm^–1^ *vs.* (b) mannosidase, (d) neuraminidase and (f) *N*-acetyl-glucosaminidase-treated cell concentration.

To further verify the application of the proposed strategy, cell surfaces glycans were regulated by treating the cells with glycan endonucleases. After MCF-7 cells were incubated with 100 U mL^–1^ mannosidase, neuraminidase, and *N*-acetyl-glucosaminidase, which can specifically cleave Man, Sia and GlcNAc, respectively, from cell surfaces at 37 °C for 30 minutes, they were subjected to the micro-competition system. For each kind of endonuclease, the change of the corresponding Raman barcode peak obtained on MGAuNS@B with the increasing *c*_cell_ was very small (Fig. 6), which was negligible and lower than that before treatment (Fig. 4c and d), indicating the specific cleavage of the glycans by the corresponding endonuclease.

**Fig. 7:**
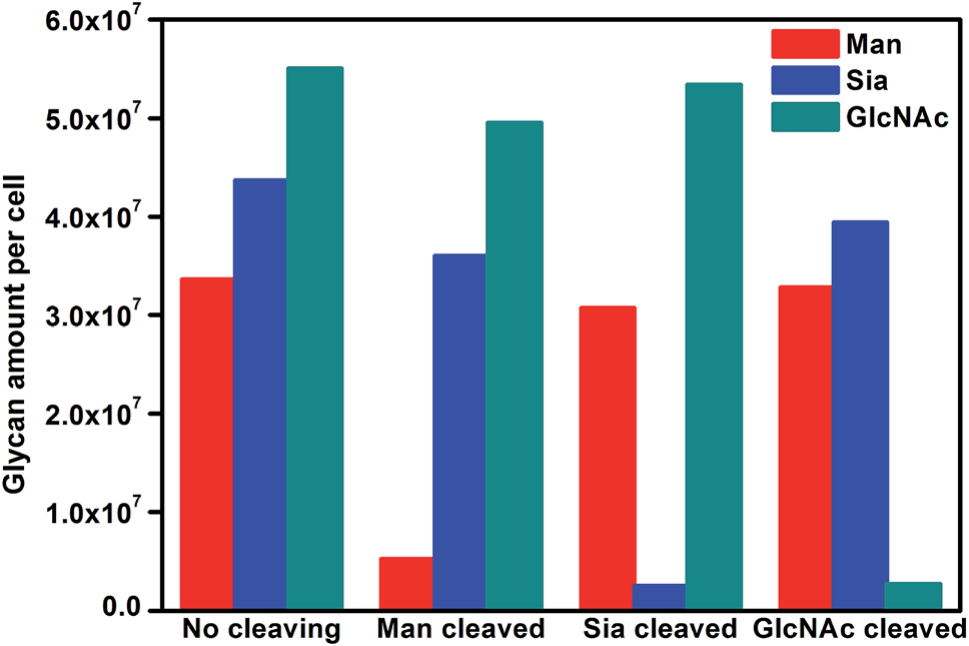
Average glycan amounts on MCF-7 cells before and after cleavage treatment with Man, Sia and GlcNAc.

From eqn (5) and the slopes of competition curves with the three kinds of treated cells for three peaks corresponding to LCA, SNA and SWGA-coded nanoprobes, the amounts of Man, Sia and GlcNAc on the corresponding glycan endonuclease-treated cells could be obtained (Fig. 7). Glycans cleaved by the corresponding glycan endonuclease showed an obvious decreased expression, while other two glycans varied imperceptibly. This also indicated the independence among the three pairs of recognition process in the micro-competition system. So the proposed strategy possessed the quantification capability of multiple glycans and could simultaneously monitor multiple glycan changes on living cell surfaces. By exploiting more Raman labels with well-spaced Raman bands,^37,38^ the multiple capability of the strategy could be expanded.

## Conclusions

A micro-competition system has been designed with multiple-polysaccharide-coated gold nanostars functionalized silica bubbles, target cells and Raman barcoded nanoprobes for quantification of multiple glycans on whole living cell surfaces. The nanoprobes can specifically recognize glycans on both natural cell and biomimic bubble surfaces and distinguish the corresponding Raman codes. The recognition in a homogeneous solution at a micron scale leads to enhanced competition efficiency. The AuNSs functionalized bubbles also endow the system with quick separation by buoyancy and sensitive detection by SERS. With this system a method for simultaneous Raman quantification of three types of glycans on intact cell surfaces has been developed. The regulation of multiple glycans on cell surfaces with glycan endonucleases has also been monitored *in situ*. The proposed strategy possesses the advantages of whole-surface accessibility, rapid detection without any cell pretreatment or labeling, convenient separation, enhanced sensitivity, high throughput and low cost. Despite the limit of available lectins and their specificity, this strategy could be expanded for other glycans or sub-types of glycans with more specific glycan–lectin interaction pairs. By combining with other biological recognition or interaction, this micro-competition system could be applied to research into the understanding of other biological interaction events.
