# Supplementary material for: Micro-competition system for Raman quantification of multiple glycans on intact cell surface
Source: Chem Sci. 2015 Apr 30;6(7):3769–74. doi: 10.1039/c5sc01031d (PMC5707490; doi:10.1039/c5sc01031d)
Supplement: Supplementary file 1 [file SC-006-C5SC01031D-s001.pdf]

Electronic Supplementary Material (ESI) for Chemical Science.
This journal is © The Royal Society of Chemistry 2015

Supplementary Information for

Micro-competition system for Raman quantification of multiple glycans on intact cell surface†

Yunlong Chen,[‡a] Lin Ding,[‡a] Junqiang Xu,[a] Wanyao Song,[a] Min Yang,[b] Junjie Hu[a] and Huangxian Ju*[a]

[a]State Key Laboratory of Analytical Chemistry for Life Science, School of Chemistry and Chemical Engineering, Nanjing University, Nanjing 210093, P.R. China. Fax: +86 25 83593593; Tel: +86 25 83593593; E-mail: hxju@nju.edu.cn

[b]Department of Pharmaceutical & Biological Chemistry, UCL School of Pharmacy, University College London, London WC1N 1AX, UK

‡ Y. L. Chen and L. Ding contributed equally to this work.

Contents

1. Materials and reagents………………………………………………………..…S2

2. Apparatus……………………………………………………………………S2

3. Experimental section…………………………………………………………S2

3.1 Preparation of nanoprobes...…………………………………………….S3

3.2 Preparation of MGAuNS@B………………………………………………S3

3.3 Standard binding curve and cell competition curve……………………S3

3.4 Monosaccharide inhibition……………………………….………………..S4

3.5 Flow cytometric analysis of glycans…………………….………………..S4

4. Supporting tables and figures……………………...…………………………S5

5. Supporting references……………………….……………………….…………S7

1. Materials and reagents

Chloroauric acid ($HAuCl_4$•$4H_2O$) and silver nitrate ($AgNO_3$) were obtained from Shanghai Chemical Reagent Company (Shanghai, China). Trisodium citrate was obtained from Sinopharm Chemical Reagent Co., Ltd. (China). S60HS silica bubbles (average diameter 30 μm, density 0.6 g/mL) were purchased from 3M Company (USA). 1-Ethyl-3-(3-dimethylaminopropyl) carbodiimide (EDC), poly(diallyldimethylammonium chloride) (PDDA), ascorbic acid, poly-L-lysine, 2-naphthol (NT), 4-aminothiophenol (ATP), 5,5'-dithiobis(2-nitrobenzoic acid) (DTNB), mannose (Man), mannan (PMan), N-acetylneuraminic acid (Sia), poly(N-acetylneuraminic acid) (PSia), N-acetylglucosamine (GlcNAc) and poly(N-acetylglucosamine) (PGlcNAc) were purchased from Sigma-Aldrich Inc. (USA). Thiol polyethylene glycol 1000 carboxyl (PEG) was obtained from Jenkem Technology Ltd. (Beijing, China). Lectins and fluorescein-labeled lectins, including *Lens culinaris* agglutinin (LCA), fluorescein-labeled LCA, *Sambucus nigra* agglutinin (SNA), fluorescein-labeled SNA, succinylated wheat germ agglutinin (SWGA) and fluorescein-labeled SWGA, were purchased from Vector Laboratories (USA). Glycan exonucleases, including mannosidase, neuraminidase and N-acetyl-glucosaminidase, were purchased from New England Biolabs (UK). Phosphate buffer saline (PBS, pH 7.4) contained 136.7 mM NaCl, 2.7 mM KCl, 8.72 mM Na_2HPO_4 and 1.41 mM KH_2PO_4. For lectin-carbohydrate interaction, 0.1 mM $CaCl_2$ and 0.1 mM $MnCl_2$ were added in PBS. All other reagents were of analytical grade. All aqueous solutions were prepared using ultrapure water (≥18 MΩ, Milli-Q, Millipore).

MCF-7 cells were purchased from KeyGen Biotech. Co. Ltd. (Nanjing, China). Cells were cultured in RPMI 1640 medium (GIBCO) supplemented with 10% fetal calf serum (FCS, Sigma), penicillin (100 μg/mL), and streptomycin (100 μg/mL) at 37 °C in a humidified atmosphere containing 5% CO_2. For quantitative use, the cells were trypsinized and washed twice with culture medium by centrifugation at 1000 rpm for 3 min. The sediment was re-suspended in PBS to obtain a homogeneous cell suspension. Cell number was determined using a Petroff-Hausser cell counter (USA).

2. Apparatus

The transmission electron microscopic (TEM) images were obtained on a JEM-2100 transmission electron microscope (JEOL Ltd., Japan). The UV-vis absorption spectra were recorded on a Nanodrop-2000C UV-vis spectrophotometer (Thermo, USA). Zeta potential analysis was performed on a Zetasizer (Nano-Z, Malvern, UK). Flow cytometric analysis was gained on a Coulter FC-500 flow cytometer (Beckman-Coulter). Raman spectra were gained on a Renishaw inVia confocal Raman microscope (Renishaw, UK) using 50 times telephoto objective and 633 nm excitation. The Raman spectra of nanoprobes on bubbles were taken by the spectral acquisition mode using static scan type at a center wavenumber of 900 cm^{-1} with 1-s exposure time and 100% laser power.

3. Experimental section

3.1 Preparation of nanoprobes

Au nanoparticles (AuNPs) were synthesized by quickly adding 1.25 mL trisodium citrate (1%) to 50 mL boiling $HAuCl_4$ solution (0.01%) under continuous stirring.[S1] The reaction mixture was stirred at 100 ^oC for 15 minutes until the color turned red or purple. The mixture was cooled down to room temperature and then stored at 4 ^oC. Before use, AuNPs were washed by centrifugation under 12000 rpm and resuspended in water. The concentration of AuNPs was determined from the UV-vis absorption spectrum.[S2]

1 μL Raman signal molecule (RSM, including NT, ATP or DTNB) of 10 mM in ethanol was added to 1 mL AuNPs solution (2 nM) containing 10 μL PEG (1 mM), which was stirred at room temperature overnight. Afterward, the solution was washed by centrifugation under 12000 rpm twice and resuspended to 1 mL in PBS buffer. 10 μL LCA, SNA or SWGA (100 μM in PBS) was one-to-one added to NT, ATP or DTNB modified AuNP solution containing 8 μL newly prepared EDC (50 mg/mL) to react for 4 hours. Three kinds of nanoprobes were obtained and washed by centrifugation under 12000 rpm twice and re-suspended to 1 mL in PBS buffer respectively.

3.2 Preparation of MGAuNS@B

AuNSs were prepared with a seed-mediated growth method.[S3] The Au seed solution was prepared by adding 7.5 mL of 1% citrate solution to 50 mL of boiling $HAuCl_4$ solution (0.04%) under vigorous stirring. After boiling for 15 min while keeping the solution volume stable, the solution was cooled and filtered by a 0.22 μm filter membrane, and then kept at 4 ^oC for long-term storage. 100 μL of the citrate-stabilized seed solution was then added to 10 mL of 0.25 mM $HAuCl_4$ (with 20 μL of 1 M HCl) in a 20 mL glass vial at room temperature under moderate stirring. After 100 μL $AgNO_3$ (2 mM) and 50 μL of ascorbic acid (100 mM) were added simultaneously, the solution was stirred for 30 s, during which its color rapidly turned from light red to blue or greenish-black, and one centrifugal wash at 3000 rpm was performed for 15 min in a 15 mL tube to obtain the precipitant. The precipitant were redispersed in water, and filtered by a 0.22 μm filter membrane to obtain AuNS solution, which was kept at 4 ^oC for long-term storage.

MGAuNSs were synthesized by adding 100 μL mixture of 10 mg/mL PMan, PSia and PGlcNAc and 100 μL citrate-stabilized seed solution in 10 mL of 0.25 mM $HAuCl_4$ (with 20 μL of 1 M HCl), and then mixing the solution with 100 μL $AgNO_3$ (2 mM) and 50 μL of ascorbic acid (100 mM) to stir for 30 s. The resulting MGAuNSs were centrifuged, redispersed in water, and filtered by a 0.22 μm filter membrane to obtain MGAuNS solution.

S60HS 3M glass bubbles of 100 mg were added to 1 mL PDDA solution (1%), and the mixture was subjected to shaking overnight on a vertical rotary mixing device to obtain positive-charged surface. The concentration of bubbles could be calculated from average diameter and density of bubbles. After the PDDA-coated bubbles were washed with water twice, excess MGAuNS solution was added and mixed on a vertical rotary mixing device until a saturation adsorption was achieved.

The obtained MGAuNS@B was blocked with BSA for 30 minutes and washed twice before use.

3.3 Standard binding curve and cell competition curve

10 μL MGAuNS@B (1×10^{7} mL^{-1}) in PBS was incubated with 90 μL mixture of nanoprobes at a series of concentrations on a vertical rotary mixing device. After 1 hour at room temperature, extra nanoprobes were removed by buoyancy separation. The nanoprobe-bound bubbles were washed with PBS twice and dried on a glass slide for Raman detection.

For cell competition analysis, 100 μL MCF-7 cells of different concentrations were incubated with 20 μL MGAuNS@B (1×10^{7} mL^{-1}) and 80 μL mixture of 2.5 nM nanoprobes. After 1-hour competitive reaction at room temperature, the bubbles were separated and washed with PBS twice and stepwise dried on a glass slide for Raman detection.

3.4 Monosaccharide inhibition

The nanoprobes (each probe of 5 nM) was incubated with 50 μM Man, Sia and GlcNAc for 2 h at room temperature, respectively, to obtain corresponding monosaccharide-inhibited probes. After the inhibited probes were purified by centrifugation under 12000 rpm for 10 min twice, respectively, and dispersed in PBS, the corresponding curves were obtained with the same procedure as that described for standard binding curve.

3.5 Flow cytometric analysis of glycans

MCF-7 cells were collected and suspended in cold PBS buffer. Fluorescein-labeled lectins of 0.1 μM were mixed with 1 mL 1×10^{6} cells/mL cell suspension, respectively. After incubation for 60 min, the cells were washed by centrifugation at 1000 rpm for 3 min twice, resuspended in 1 mL PBS, and assayed by flow cytometry. The MCF-7 cells without any treatment were used as the negative control for estimation of autofluorescence.

4. Supporting tables and figures

Table S1 Lectins and their glycan-binding specificity[a] used in this work.

Lectin	Source	Binding specificity[b]	Raman barcode
LCA	*Lens culinaris* (lentil) seeds	αMan, αGlc	NT
SNA	*Sambucus nigra* (Elderberry) bark	Siaα6Gal/GalNAc	ATP
SWGA	*Triticum vulgaris* (wheat germ)	GlcNAc	DTNB

[a]Ref: Vector Laboratories Web site (http://www.vectorlabs.com/data/protocols/K4−K7.pdf).

[b]Glc, Gal and GalNAc represent glucose, galactose and N-Acetylgalactosamine, respectively.

Figure S1 UV-vis spectra of AuNPs, three lectins and corresponding Au-RSM/PEG and nanoprobes.

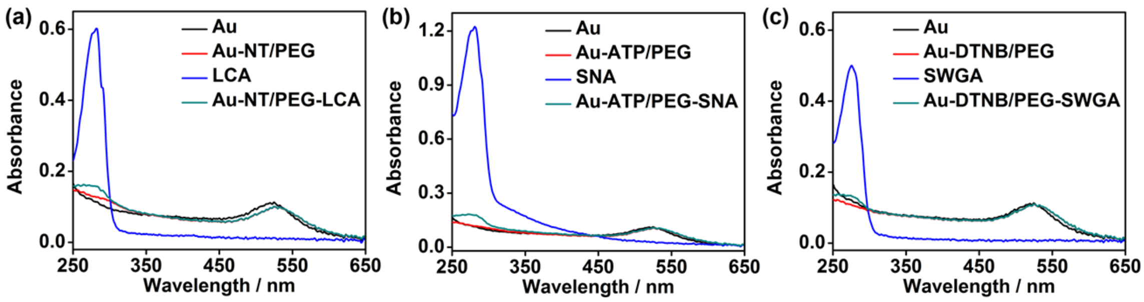

Figure S2 TEM images of a) AuNS and b) MGAuNS. c) Zeta potentials of AuNS and MGAuNS. d) Raman spectra of three kinds of RSMs on AuNS and MGAuNS.

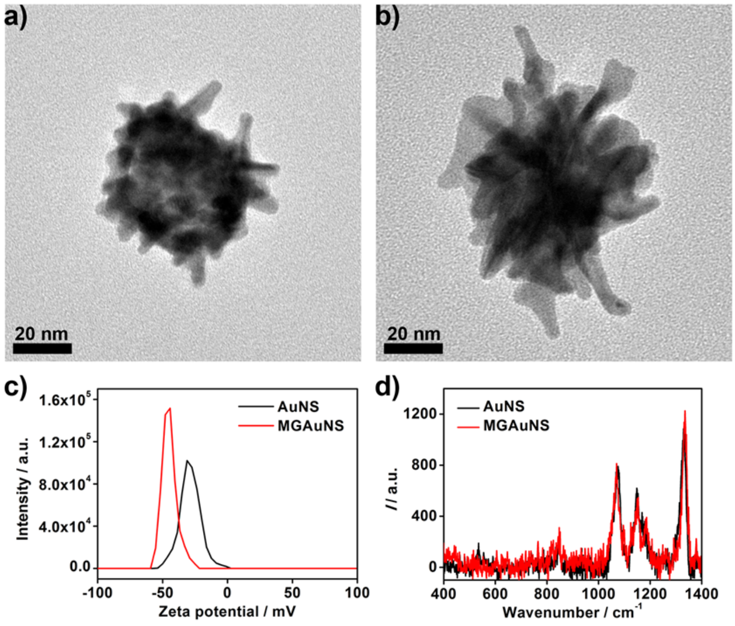

Figure S3 Raman spectra of AuNS@B, PManAuNS@B, PSiaAuNS@B, and PGlcNAcAuNS@B after incubation with the mixture of LCA, SNA and SWGA labeled nanoprobes, respectively.

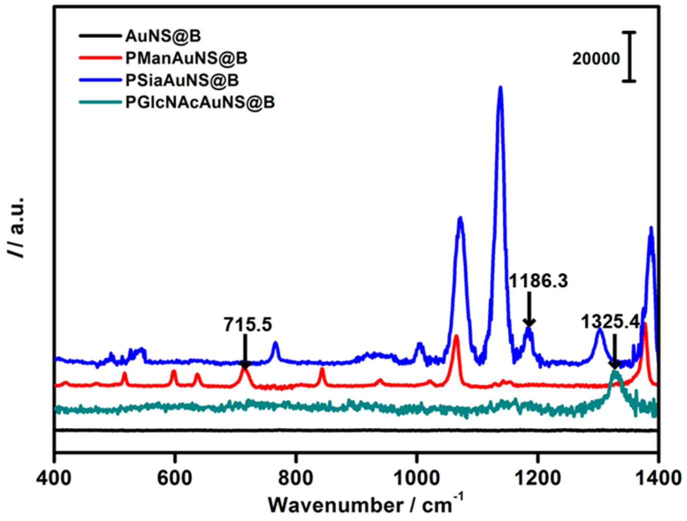

Figure S4 Raman spectra of MGAuNS@B after incubation with the mixture of Man a), Sia c) and GlcNAc e) pre-inhibited nanoprobes at different concentrations, and plots of Raman intensity at 715.3, 1187.2 and 1325.1 cm^{-1} *vs.* concentrations of Man b), Sia d) and GlcNAc f) pre-inhibited nanoprobes that were labeled with LCA, SNA and SWGA respectively.

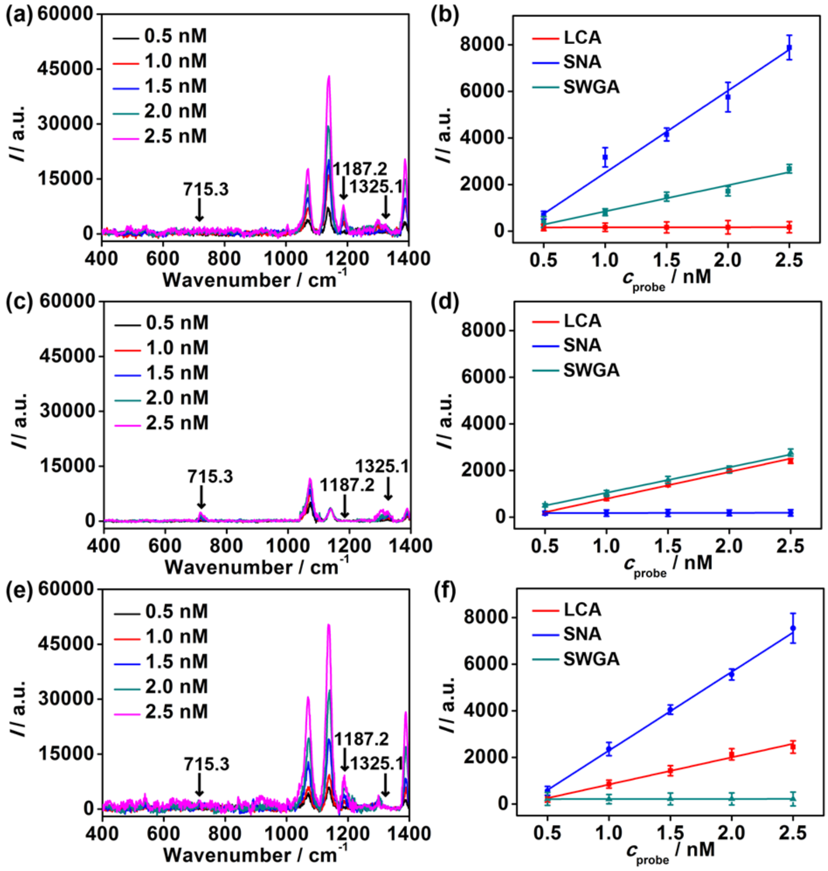

Figure S5 Raman spectra of MGAuNS@B after incubation with the mixture of three nanoprobes for different incubation times in a) absence and c) presence of cells, and b,d) corresponding plots of Raman intensity at 1069.7 cm^{-1} *vs.* incubation time.

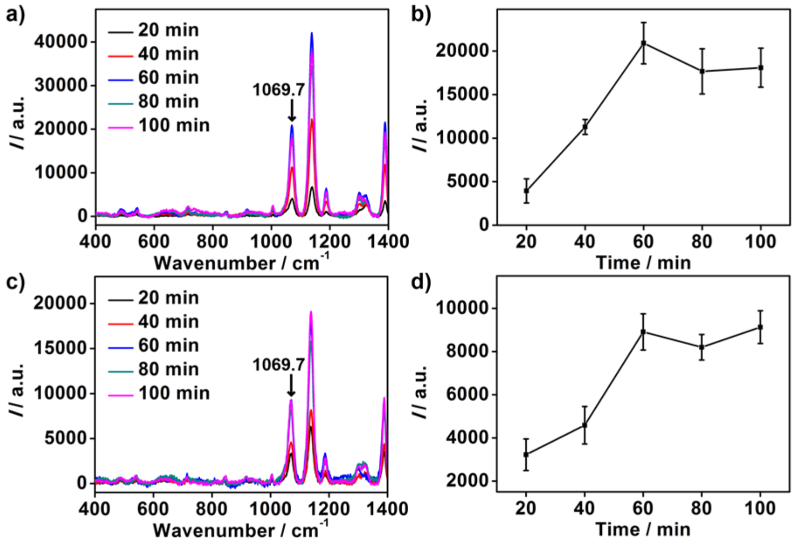

Figure S6 Flow cytometric analysis of MCF-7 cells after incubation with fluorescein-labeled LCA, SNA and SWGA.

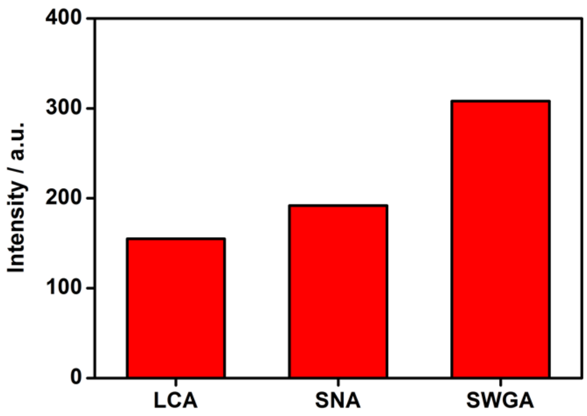

5. Supporting references

[S1] G. Frens, *Nat. Phys. Sci.,* 1973, **241**, 20-22.

[S2] W. Haiss, N. T. K. Thanh, J. Aveyard, and D. G. Fernig, *Anal. Chem.,* 2007, **79**, 4215-4221.

[S3] H. Yuan, C. G. Khoury, H. Hwang, C. M. Wilson, G. A. Grant, and T. Vo-Dinh, *Nanotechnology,* 2012, **23**, 075102.
